# Natural Mating Differentially Triggers Expression of Glucocorticoid Receptor (NR3C1)-Related Genes in the Preovulatory Porcine Female Reproductive Tract

**DOI:** 10.3390/ijms21124437

**Published:** 2020-06-22

**Authors:** Mateo Ruiz-Conca, Jaume Gardela, Cristina Alicia Martínez, Dominic Wright, Manel López-Bejar, Heriberto Rodríguez-Martínez, Manuel Álvarez-Rodríguez

**Affiliations:** 1 Department Biomedical and Clinical Sciences (BKV), BKH/OG, Linköping University, 58185 Linköping, Sweden; mateo.ruiz@uab.cat (M.R.-C.); jaume.gardela@uab.cat (J.G.); cristina.martinez-serrano@liu.se (C.A.M.); heriberto.rodriguez-martinez@liu.se (H.R.-M.); 2 Department of Animal Health and Anatomy, Veterinary Faculty, Universitat Autònoma de Barcelona, 08193 Cerdanyola del Vallès, Spain; manel.lopez.bejar@uab.cat; 3 Department of Physics, Chemistry and Biology, Faculty of Science and Engineering; Linköping University, 58183 Linköping, Sweden; dominic.wright@liu.se; 4 College of Veterinary Medicine, Western University of Health Sciences, Pomona, CA 91766, USA

**Keywords:** transcriptomics, microarrays, spermatozoa, mating, glucocorticoid, *FKBP5*, *FKBP4*, *NR3C1*, female reproductive tract, pig

## Abstract

Mating initiates dynamic modifications of gene transcription in the female reproductive tract, preparing the female for fertilization and pregnancy. Glucocorticoid signaling is essential for the homeostasis of mammalian physiological functions. This complex glucocorticoid regulation is mediated through the glucocorticoid receptor, also known as nuclear receptor subfamily 3 group C member 1 (NR3C1/GR) and related genes, like 11β-hydroxysteroid dehydrogenases (HSD11Bs) and the FK506-binding immunophilins, FKBP5 and FKBP4. This study tested the transcriptome changes in NR3C1/GR regulation in response to natural mating and/or cervical deposition of the sperm-peak ejaculate fraction collected using the gloved-hand method (semen or only its seminal plasma), in the preovulatory pig reproductive tract (cervix to infundibulum, 24 h after mating/insemination/infusion treatments). Porcine cDNA microarrays revealed 22 *NR3C1*-related transcripts, and changes in gene expression were triggered by all treatments, with natural mating showing the largest differences, including *NR3C1*, *FKBP5, FKBP4,* hydroxysteroid 11-beta dehydrogenase 1 and 2 (*HSD11B1, HSD11B2),* and the signal transducer and activator of transcription 5A (*STAT5A*). Our data suggest that natural mating induces expression changes that might promote a reduction of the cortisol action in the oviductal sperm reservoir. Together with the STAT-mediated downregulation of cytokine immune actions, this reduction may prevent harmful effects by promoting tolerance towards the spermatozoa stored in the oviduct and perhaps elicit spermatozoa activation and detachment after ovulation.

## 1. Introduction

Glucocorticoids (GCs) are steroid hormones essential for adaptation to stress, behavior, and reproduction. GC release is under circadian/ultradian pulsatile control of the hypothalamic–pituitary–adrenal (HPA) axis and its regulation is absolutely necessary for animal homeostasis. Undeniable new evidence suggests a crucial role of GCs during different reproductive steps, where they display central and peripheral regulation [1]. The balance between high and low levels of GCs determines whether their biological activity mediates the correct functions or causes pathology. In this sense, it has long been assumed that GCs act negatively on reproductive function due to their role in chronic stress physiology [2]. However, while high chronic levels of the hormones in response to stress are pathological and affect fertility, basal levels of GCs are also essential for normal reproduction [1]. For instance, GCs undergo a significant rise prior to ovulation in most mammalian species, and although their increase may lead to impairment of their reproductive function [3], their presence is essential during embryonic/fetal development, parturition, and lactation [1,4,5]. GCs are also relevant for male fertility, and while GC-exposure drives the inhibition of steroidogenesis in the testis and apoptosis of germ cells [6], its deficiency impairs testicular function [5,7]. Although many studies address their influence in animal reproduction [8,9,10,11], the mechanisms by which GCs participate in energetically-demanding processes [12] (including sperm transport and storage in the female reproductive tract, the modulation of the immune response, and also their involvement in gene transcription signaling) are still not well known.

Reproductive events that are initiated by either natural mating or artificial insemination (AI), such as sperm transport, sperm storage, fertilization, and the cascade of embryonic/fetal processes associated with implantation, placentation, cervical ripening, and final delivery, are all accompanied by inflammatory, immunological, and transcriptional responses in the reproductive tract. These events are often modulated by GCs, whose regulatory signaling action seem to be mediated by one particular receptor, the glucocorticoid receptor. This receptor, also known as the nuclear receptor subfamily 3 group C member 1 (NR3C1/GR), binds to the GC forming a complex that is translocated to the nucleus of the cells to modify gene transcription [13]. This translocation is done with the aid of partner molecules [10,14,15], including the peptidyl-prolyl cis/trans isomerase FK506-binding proteins (FKBP family proteins), which may regulate this translocation [16]. Thus, cortisol–glucocorticoid receptor complexes are responsible for the activation or repression of transcription of target genes (up to 10–20% of the whole genome in humans) [17,18] triggering, in turn, cascades with pleiotropic implications, that include reproductive, immune, and transgenerational effects [8,19].

In this regard, natural mating shapes reproductive physiology in a variety of different species due to a combination of the sensorial stimulation produced by penile buffeting, the presence of spermatozoa and/or the contact with seminal plasma (SP) [20,21,22]. The aforementioned mating factors seem to change gene and protein expression post-coitus, consequently modulating uterine and oviductal functions [23]. Such modifications elicited in the reproductive tract help to create a suitable environment necessary for sperm storage [24], gamete transport, pre-implantation development, increased angiogenesis, and also changes in the immune system pattern [25,26], including the attainment of a status of tolerance to foreign proteins and cells. Although GC signaling is essential for the establishment and maintenance of fertility [1], the up-stream triggers causing the modifications of *NR3C1*-related genes after natural mating or insemination in the preovulatory phase are yet to be determined.

Therefore, the present study tested the hypothesis that (i) natural mating, (ii) the cervical deposition via AI of the sperm-peak ejaculate fraction, and (iii) AI of the sperm-free SP, all equally affect the expression of 22 genes involved in NR3C1/GR regulation in the preovulatory pig reproductive tract, 24 h after treatment. These treatments were performed on samples of mature fertile boars (*n* = 5) collected using the gloved-hand method.

## 2. Results

### 2.1. Gene Ontology of the Genes Related to the Glucocorticoid Receptor NR3C1

**Figure 1:**
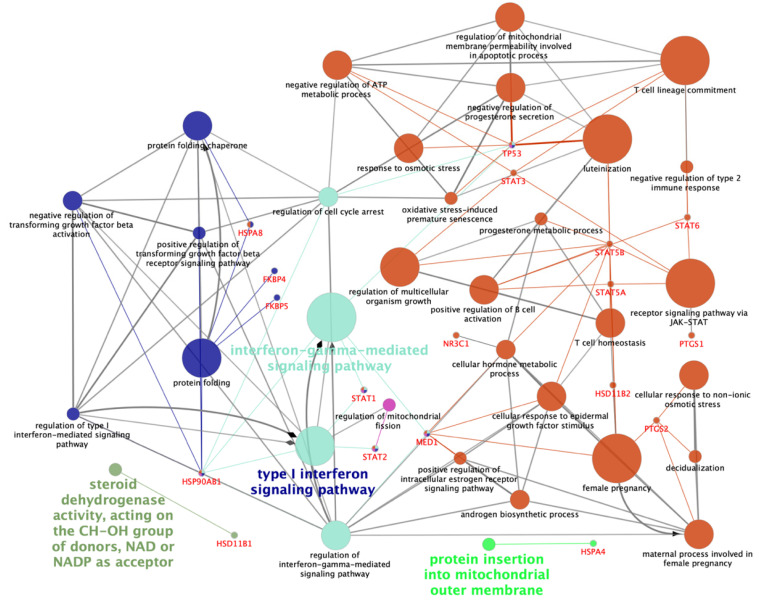
Schematic representation of altered transcripts of interest among all tissues and treatments. The analysis of over-represented functional categories was performed using the Cytoscape v3.0.0 application ClueGo v2.0.3. The following databases were used: Gene Ontology (GO) subgroups biological process which is shown as circles. Terms are functionally grouped based on shared genes (kappa score) and are shown in different colors. The size of the nodes indicates the degree of significance, where the biggest nodes correspond to the highest significance. The most significant term defines the name of the group. The following ClueGo parameters were used: biological process database (BP; date: 19.05.2020); GO tree levels, 2–4 (first level = 0); minimum number of genes, 1; minimum percentage of genes, 1.2; GO term fusion; GO term connection restriction (kappa score), 0.4; GO term grouping, initial group size of 2 and 50% for group merge. The resulting network representation was manually rearranged after removing unnecessary terms. HSD11B1 and HSD11B2 (hydroxysteroid 11-beta dehydrogenase 1 and 2), HSPA4, HSPA8 and HSP90AB1 (heat shock proteins 4, 8 and 90 alpha family class B member 1), FKBP5 and FKBP4 (FK506-binding prolyl isomerase 5 and 4), MED1 (mediator complex subunit 1), NR3C1 (nuclear receptor subfamily 3 group C member 1), PTGS1 and PTGS2 (prostaglandin-endoperoxide synthase 1 and 2), STAT1, STAT2, STAT3, STAT5A, STAT5B and STAT6 (signal transducer and activator of transcription 1, 2, 3, 5A, 5B, and 6), TP53 (tumor protein 53).

The 22 *NR3C1*-related genes of interest, directly or indirectly engaged in glucocorticoid receptor NR3C1 action and being potentially involved in reproductive functions and signaling (Figure 1), were analyzed. Besides *NR3C1*, these genes included the following: hydroxysteroid 11-beta dehydrogenase 1 and 2 (*HSD11B1* and *HSD11B2*), the FK506-binding prolyl isomerase 5 and 4 (*FKBP5* and *FKBP4*), the prostaglandin-endoperoxide synthase 1 and 2 (*PTGS1* and *PTGS2*), the phospholipase A2 group IVB (*PLA2G4B*), the insulin-like growth factor binding protein 1 (*IGFBP1*), the heat shock protein family A (*HSP70*) member 8 (*HSPA8*), the signal transducer and activator of transcription 1, 2, 3, 5A, 5B, and 6 (*STAT1, STAT2, STAT3, STAT5A*, *STAT5B,* and *STAT6*), the tumor protein P53 (*TP53*), the mediator complex subunit 1 and 14 (*MED1* and *MED14*), the heat shock protein family A (*HSP70*) member 4 and member-4-like (*HSPA4* and *HSPA4L*), and heat shock protein 90 alpha family class B member 1 (*HSP90AB1*).

### 2.2. Natural Mating and AI of Semen Components Altered the Expression of Genes Related to the Glucocorticoid Receptor NR3C1

**Figure 2:**
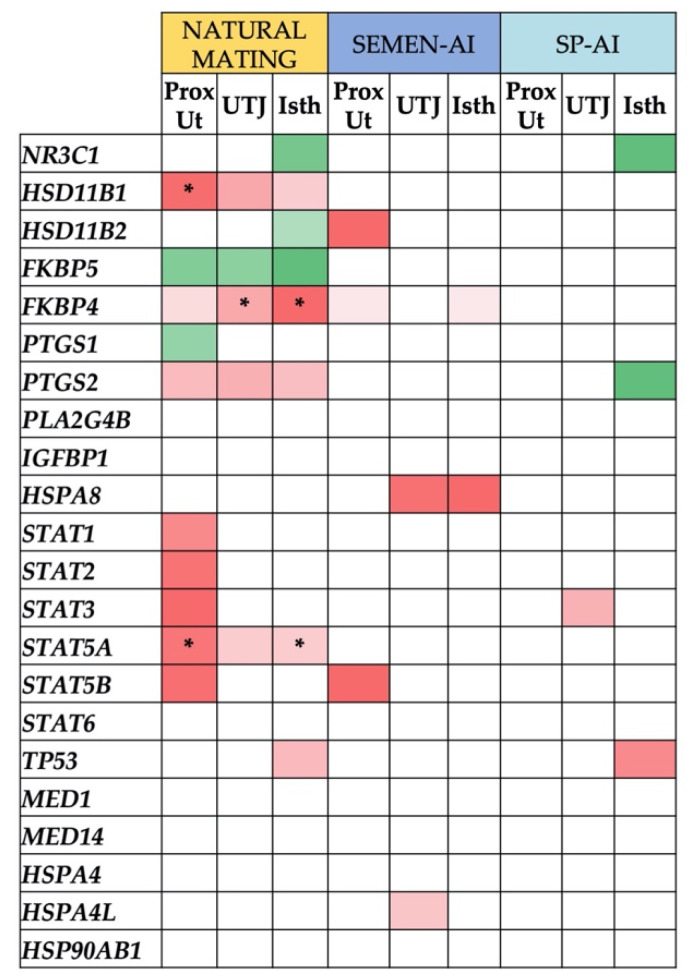
Differentially-expressed genes (DEGs) in proximal uterus (ProxUt), utero-tubal junction (UTJ), and isthmus (Isth) tissues, ordered by treatments (natural mating, semen-artificial insemination (AI), or sperm-free (SP)-AI). Upregulated genes (*p* < 0.05) are marked in green, while downregulated genes (*p* < 0.05) are shown in red. Color grading is displayed, ranging from 1 (upregulated) to −1 (downregulated), separately for every treatment. False discovery rates (*q* < 0.05) are noted with an asterisk.

Figure 2 shows the genes related to the glucocorticoid hormone receptor NR3C1 that were differentially expressed (*p* < 0.05) in the proximal uterus (ProxUt), utero-tubal junction (UTJ), and isthmus (Isth) of the sow reproductive tract 24 h after (i) natural mating, (ii) cervical insemination with the first portion of sperm-rich fraction (semen-AI), or (iii) cervical infusion with the sperm-free seminal plasma of this portion (SP-AI). Interestingly, some of the genes were similarly downregulated (*HSD11B1, FKBP4, PTGS2,* and *STAT5A*) or upregulated (*FKBP5*) in these three genital compartments, considered the most biologically relevant during the preovulatory phase [27,28]. The overall differentially-expressed genes, from cervix (Cvx) to infundibulum (Inf) are represented in Supplementary Figure S1.

**Figure 3:**
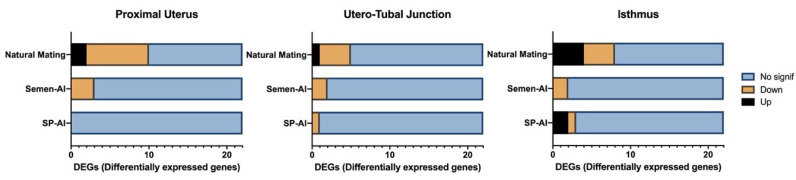
Number of differentially-expressed genes (DEGs) present in proximal uterus, utero-tubal junction, and isthmus tissues. Upregulated genes (*p* < 0.05) are represented in black and downregulated genes (*p* < 0.05) in orange color. Non-significant genes are represented in blue.

Natural mating (Figure 3) was, by far, the treatment that caused the largest changes to gene expression. In mating, 23 differential expression changes were registered (10 in ProxUt, five in UTJ, and eight in Isth), with 17 out of the 23 affected genes being downregulated (73.9%) in the reproductive tract segments (*p* < 0.05). Remarkably, all significant genes identified using an FDR (false discovery rate)-corrected threshold (*q* < 0.05) were solely found in the natural mating treatment group (five differential expression changes). In contrast, in the semen-AI group only seven differential expression changes were found (*p* < 0.05), all of them being downregulation. Regarding the seminal plasma treatment (SP-AI), there were a total of four differential expression changes (*p* < 0.05), two of them being downregulated (50%).

Thus, in ProxUt tissue (Figure 2), natural mating upregulated *FKBP5* and *PTGS1* but downregulated *FKBP4, PTGS2, HSD11B1* (*q* < 0.05), *STAT1, STAT2, STAT3, STAT5A* (*q* < 0.05), and *STAT5B* (*p* < 0.05). Semen-AI downregulated *HSD11B2, FKBP4,* and *STAT5B* (*p* < 0.05) while SP-AI infusion resulted in no differentially-expressed genes (DEGs).

In the UTJ (Figure 2), considered the main functional oviductal sperm reservoir in pigs, natural mating also upregulated the *FKBP5* gene (*p* < 0.05) while downregulating *FKBP4* (*q* < 0.05), *HSD11B1*, *PTGS2,* and *STAT5A* (*p* < 0.05). The other treatments using AI downregulated (*p* < 0.05) *HSPA8* and *HSPA4L* in semen-AI, and *STAT3* in the case of SP-AI.

In the Isth tissue (Figure 2), natural mating upregulated *FKBP5, NR3C1,* and *HSD11B2* (*p* < 0.05), while downregulating *FKBP4* (*q* < 0.05), *HSD11B1, PTGS2, STAT5A* (*q* < 0.05), and *TP53* (*p* < 0.05). The semen-AI treatment downregulated *FKBP4* and *HSPA8* (*p* < 0.05) while the SP-AI infusion treatment upregulated *NR3C1* and *PTGS2* (*p* < 0.05) and downregulated *TP53* (*p* < 0.05).

Regarding the rest of the tissues tested, in the Cvx, only sperm-containing treatments induced a change in gene expression, downregulating all genes (*p* < 0.05). Natural mating induced downregulation of *FKBP4, HSD11B1, PTGS2, STAT1, STAT2, STAT5A, TP53,* and *HSPA4* (*p* < 0.05) while semen-AI downregulated *HSPA4L* (*p* < 0.05). In the distal uterus (DistUt), natural mating induced a similar expression pattern to that seen in the ProxUt tissue (7/3 and 8/2 down/upregulated genes, respectively). Here, *FKBP5, HSP4L* (*q* < 0.05), and *HSP90AB1* were upregulated while *FKBP4, HSD11B1* (*q* < 0.05), *STAT1, STAT2, STAT3, STAT5A* (*q* < 0.05), and *STAT6* (*p* < 0.05) were downregulated. Interestingly, both semen-AI (first portion of sperm-rich fraction) and its sperm-free seminal plasma (SP-AI) treatments, induced *PTGS1*-downregulation in the DistUt (*p* < 0.05). The seminal plasma treatment (SP-AI) induced upregulation of *HSPA4L* (*p* < 0.05). The ampulla (Amp) and Inf were the tissues showing most DEGs. Natural mating displayed a similar expression pattern in the two tissues with *FKBP4* (*q* < 0.05), *HSPA8, HSPA4L, HSP90AB1,* and *MED14* being downregulated (*p* < 0.05) and *HSD11B2* (*q* < 0.05 in Inf), *STAT3* (*q* < 0.05 in Amp), and *STAT6* (*q* < 0.05 in Inf), being upregulated (*p* < 0.05). The *PLA2G4B* and *PTGS1* genes were upregulated in Amp in natural mating (*p* < 0.05). Additionally, in the Amp, *PLA2G4B* was also upregulated by semen-AI and SP-AI treatments (*p* < 0.05). Ampullar downregulation of *MED1* was induced by natural mating and semen-AI treatments (*p* < 0.05). Semen-AI induced downregulation of *HSPA8, HSPA4,* and *STAT1* and upregulated *STAT6* (*p* < 0.05) while the SP-AI treatment only downregulated *STAT1* (*p* < 0.05). In the Inf, the sperm-containing treatments (natural mating and semen-AI) upregulated *NR3C1* (*p* < 0.05) while the semen-AI treatment also upregulated *PLA2G4B* (*p* < 0.05). Similar to the other tissues, *STAT5A* was downregulated by natural mating (*p* < 0.05) while *HSPA8, FKPB4,* and *HSPA4* were downregulated by semen-AI and natural mating (*p* < 0.05). *STAT5A* and *TP53* were downregulated by SP-AI (*p* < 0.05).

Additionally, the principal component analysis (PCA, [29]), showed that in the ProxUt, the first principal component (PC1) explained 34.6% and the second principal component (PC2) 24% of the total variance (*n* = 16). For the UTJ, PC1 and PC2 explained 25.7% and 22.2%, respectively (*n* = 16). In the case of Isth, PC1 explained 28% and PC2 explained 18% (*n* = 16) (see Supplementary Figure S2). A heat map representation for the ProxUt, UTJ, and Isth tissues was performed to aid visualization of group separation. Data was clustered using correlation distance and average linkage (see Supplementary Figure S3).

### 2.3. KEGG Pathways Analysis

Differentially-expressed genes were annotated into different biological pathways by using the official names of the genes and the *Sus scrofa* annotation in the Kyoto Encyclopedia of Genes and Genomes (KEGG) pathways database [30]. The KEGG pathways enabled the analysis and organization of the detected genes according to their signaling pathways. The data was organized by treatment, and by the total number of genes analyzed (22), 21 genes (95%) were represented in the natural mating group (Supplementary Table S1), 12 genes (55%) in the semen-AI group (Supplementary Table S2), and nine genes (41%) in the SP-AI group (Supplementary Table S3). The most enriched pathways were the Janus kinase/signal transducers and the activators of transcription (JAK/STAT) signaling (ssc0463). Several other important pathways were also identified, including pathways such as Th17 differentiation (ssc04659), Th1 and Th2 differentiation (ssc04658), and estrogen signaling (ssc04915).

### 2.4. PANTHER Gene Ontology Analysis

**Figure 4:**
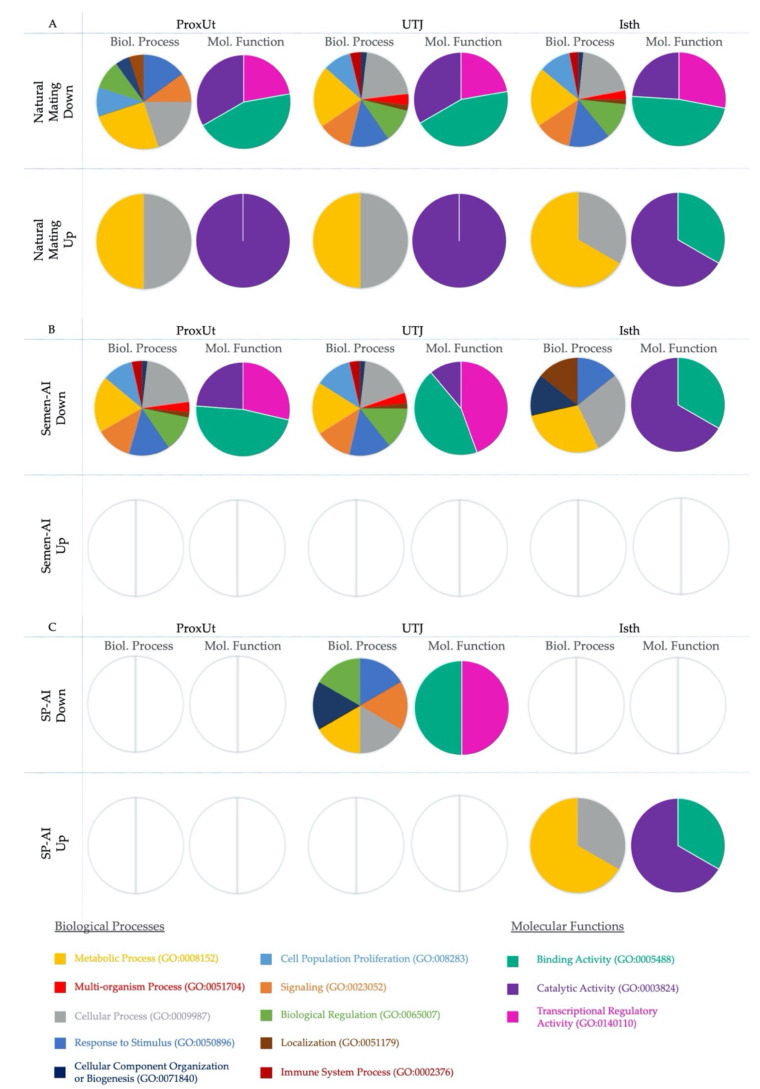
(**A**–**C**). Differentially-expressed genes (DEGs) classified in PANTHER according to biological processes and molecular functions in natural mating (**A**), semen-AI (**B**), and SP-AI (**C**). Rows display the treatment and the up- or downregulation of the analyzed DEGs (*p* < 0.05). Proximal uterus (ProxUt), utero-tubal junction (UTJ), and isthmus (Isth) tissues are shown in the columns. We have specified if the representations correspond to biological processes (Biol. Process; 1st, 3rd, and 5th column) or molecular functions (Mol. Function; 2nd, 4th, and 6th column) are depicted in colors. Empty charts indicate where no matches were found for those DEGs, or if these DEGs were absent from that experimental group.

Figure 4 shows (in separate columns) the results of the analyses regarding biological processes and molecular functions using the PANTHER (Protein Analysis Through Evolutionary Relationships) Classification System for gene ontology (GO) [31]. The results were subdivided by treatment, with the ProxUt, UTJ, and Isth tissues treated with natural mating shown in Figure 4A, the semen-AI results shown in Figure 4B, and the SP-AI treatment results shown in Figure 4C. The results of the biological processes after natural mating analysis (Figure 4A) showed that downregulated DEGs display biological processes mainly focused on metabolic (GO:0008152) and cellular process (GO:0009987) followed by response to stimulus (GO:0050896). Attending to tissue differences, importance of localization (GO:0051179) and cellular component organization and biogenesis (GO:0071840) was observed in Cvx, DistUt, and ProxUt tissues, while immune system processes (GO:0002376) were only represented in the tissues from UTJ to Inf. In the case of upregulated DEGs, the representation of biological processes is similar to the response displayed by downregulated genes, but it also seemed to display a particular pattern of metabolic and cellular process in the ProxUt, UTJ, and Isth. In the case of the semen-AI PANTHER results (Figure 4B), downregulated genes showed an interesting pattern focused in the Isth, where cellular component organization and biogenesis, and cellular process and localization seem to be particularly promoted. Regarding seminal plasma influence (Figure 4C), the data available only found significant biological processes for a few tissues, but upregulated DEGs seemed to display a similar pattern focused on metabolic and cellular processes in the Isth, while downregulated DEGs displayed a range of processes that were largely different to the sperm-containing treatments. Regarding the molecular functions data, binding activity (GO:0005488) seems to be the main function altered in all groups. As well as this, there was an apparent higher transcriptional regulatory activity (GO:0140110) in Amp and Inf tissues, while the catalytic activity (GO:0003824) seems to be more present in Isth than in other tissues, at least in the AI treatments.

## 3. Discussion

In the present study, we analyzed preovulatory expression changes of the glucocorticoid receptor NR3C1/GR-related genes in tissues collected from the porcine reproductive tract 24 h after natural mating or intra-cervical insemination/infusion of the first portion of sperm-peak ejaculate or its sperm-free seminal plasma. In general, all treatments tested induced gene expression changes, but natural mating clearly induced the most changes in gene expression as compared to the other tested treatments. Insemination of the peak sperm-rich portion (semen-AI) induced a greater response than its seminal-plasma counterpart (SP-AI), presumably due to the effect exerted by the relevant presence of spermatozoa [32]. Almost all genes analyzed (21 out of 22) displayed changes, and more than 60% of the total DEGs for all treatments were achieved by mating. Natural mating might intrinsically display local and systemic effects, as the sensory stimulation produced by penis buffeting affecting the uterus and oviduct motility [21], or the responses after the semen contact the epithelial lining. Mating influences oviduct motility and secretion due to the expression of genes associated with differentiation of oviductal cells, sperm storage, angiogenesis, gamete transport, or the immune system [25]. Here, natural mating triggered the differential expression of *NR3C1*, but also of related genes related to the regulation of GC in the reproductive tract during the preovulatory phase. Natural mating modulates gene expression along the entire reproductive tract, including the functional reservoir, inducing changes in several species [33,34,35]. It also elicits specific responses from the different *NR3C1*-related genes perhaps oriented to the reduction of cortisol [36]. This response may be preventing potentially harmful effects on spermatozoa by the female immune response, thus GCs may promote the development of tolerance towards spermatozoa in the oviduct [36]. 

It has been suggested that glucocorticoids may take part in the intrauterine regulations during early pregnancy in bovines [11]. In ruminants, previous studies indicated a relevant involvement of glucocorticoids in the regulation of uterus [37]. To date, studies in pig are scarce. What is known is that the control of the levels of active cortisol and the interaction with its receptor, the NR3C1 glucocorticoid receptor, are mediated by the expression of *HSD11B1* and *HSD11B2* conforming to the so-call pre-receptor ligand metabolism [38], while the glucocorticoid receptor action is actively regulated by, among other factors, the FKBP genes, *FKBP4* and *FKBP5*.

The potential role for the genes *HSD11B1* and *HSD11B2* is modulating GC levels in the genital tract [39]. These genes encode for the hydroxysteroid dehydrogenases, responsible for the bidirectional oxidation of cortisol into cortisone (11β-HSD2) and cortisone reduction to active cortisol (11β-HSD1) [40]. Natural mating causes upregulation of *HSD11B2* in tissues from isthmus to infundibulum. This may indicate that cortisol transformation into inactive cortisone in the preovulatory pig oviduct is promoted at transcriptomic level. Meanwhile, the gene *HSD11B1,* responsible for cortisone conversion into active cortisol [39,40], was downregulated in all reproductive tract segments from the cervix to the isthmus. Interestingly, in pigs, high 11β-HSD1-mediated cortisol production activity has been related to the inhibition of porcine oocyte maturation [41,42,43] in contrast to species such as the cow, where it appears to be promoted after ovulation [13,44,45] and is also beneficial for oocyte maturation and fertilization, at least in vitro [44,46,47]. Furthermore, in equine species, cortisol does not affect oocyte maturation in vitro [48]. The direct effect of GCs in mammalian species appears to vary greatly, implying that GC-sensitivity and regulation might be species-specific, and in part driven by diverse ratios of HSD11B2/1 [41].

Even though the active cortisol ligand availability is relatively restricted during follicular maturation in part due to the action of HSD11B2 [5], we found the glucocorticoid receptor activated in oviductal tissues. The glucocorticoid receptor gene (*NR3C1*) is a master transcriptional regulator that plays a key role in a large number of vertebrate physiological functions such as stress signaling, the immune system, behavior, metabolic activity, and also reproductive events [1]. The expression of *NR3C1* has been described in the female reproductive tract in human [49], ovine [10,50], and bovine endometrium and oviduct [9,11,51]. Our results show that *NR3C1* is upregulated by natural mating in the isthmus and infundibulum, while semen-AI only upregulated the gene in the infundibulum, and the SP-AI treatment only upregulated the gene in the isthmus. The fact that the expression in the isthmus is triggered by both natural mating and also by the first portion of the seminal plasma (SP-AI) may indicate an effect of the complex seminal plasma [52]. Glucocorticoids levels are known to increase shortly after ovulation to exert anti-inflammatory actions, helping after the follicle rupture, maintaining of the corpus luteum, and contributing to steroidogenesis, where glucocorticoids stimulate the pregnenolone to progesterone conversion [5,38]. In that sense, it could be possible that glucocorticoid receptor expression may be promoted after mating for the upcoming events. Previous studies in *NR3C1* knock-out rodents, found an exaggerated inflammatory response, aberrant immunomodulation, and immune cell recruitment [8], even linked to transgenerational effects [19] and important reproductive pathologies [53]. Moreover, there is another receptor with a high degree of sequence homology with NR3C1 and a high, but also promiscuous, affinity for glucocorticoid binding, the mineralocorticoid receptor (*NR3C2*) [54]. Despite their similarity, both receptors display very different transcriptional and physiological outcomes upon their activation, and we did not find *NR3C2* expression in our study.

On the other hand, the genes *FKBP4* and *FKBP5* are importantly involved in the regulation of NR3C1 signaling [55,56]. FKBP51 immunophilin (*FKBP5*) and other cofactors are bound when the receptor is cytosol-located and inactive, resulting in a complex [57,58]. After GC binding, FKBP51 is interchanged with FKBP52 (*FKBP4*) [59,60]. One of the functions of FKBP52 consists of translocating the complex to the nucleus [59], where NR3C1 complex binds to the glucocorticoid response elements, inducing activation or repression of an important number of genes [1,61]. As a part of this mechanism of action, NR3C1 also exerts a rapid induction of *FKPB5* transcription, generating an ultra-short negative feedback loop that hinders the translocation to the nucleus [60], blocking NR3C1-mediated actions [57,62,63]. Thus, while *FKBP5* seems to block GC action by reducing receptor affinity for the ligands, *FKBP4* increases its ligand affinity, allowing translocation of the receptor to the nucleus [60,64]. This may be in agreement, at least at genomic level, with our results in the uterus, UTJ, and isthmus, where we find an upregulation of the *FKBP5* gene and a downregulation of the *FKBP4* gene. The overexpression of *FKBP5* has been mooted as being responsible for the adaptive mechanism developed in New World primates to control their high levels of circulating GC via immunophilins [65]. In contrast, the constant downregulation observed in *FKBP4* expression in both sperm-containing treatments may limit the NR3C1 complex translocating to the nucleus, consequently reducing the detrimental effects of immune attack to preovulatory-present spermatozoa. Overall, the putative roles of *FKBP5* in relation to the biological activity of NF-κB (nuclear factor kappa-light chain-enhancer of activated B cells) [66,67], might also prevent aberrant immune activation that would affect sperm function and survival in the oviduct.

Other genes involved in GC regulation are *PTGS1* and *PTGS2*, encoding for constitutive and inducible prostaglandin synthase enzymes (PTGS1 or COX-1 and PTGS2 or COX-2, respectively), and *PLA2G4B* that encodes for phospholipase A2 that previously transforms membrane phospholipids in arachidonic acid (ARA). This ARA is subsequently converted by PTGS1/PTGS2 into PGH2, a precursor for active prostaglandins [68,69]. We found that natural mating downregulated *PTGS2* and *HSD11B1* in the same tissues, from the cervix to the isthmus. *PTGS2* upregulation is linked to 11β-HSD1 stimulation, and *PTGS2*-derived prostaglandins seem to influence cortisol availability to NR3C1 [10]. Thus, our results could indicate that natural mating leads to a downregulation of *PTGS2* in the reproductive tract from cervix to isthmus, while upregulating *PTGS1* in some tissues. Recent evidence of a specific *PTGS1*–*PTGS2* compensation mechanism involving these two genes indicates they may be responsible for prostaglandin synthesis in reproductive tissues [70]. Also, the observed increase in *PLA24GB* gene expression, responsible for the ARA precursor of prostaglandins, in the ampulla by natural mating as well as by AI-treatments (semen-AI and SP-AI) might be related to the expression of the oviductal phospholipase A2 gene (*PLA2G4B*) close to the fertilization site and may be specifically stimulated by the first seminal plasma portion.

Interestingly, the *NR3C1*-related heat shock proteins (HSPs) were downregulated by both natural mating and AI using sperm-rich fraction components. *HSPA8* (both mating and semen-AI), *HSPA4L* (mating), *HSP90AB1* (mating), and *HSPA4* (semen-AI) expression changes were triggered in exactly the same oviductal tissues (Amp and Inf). Thus, we suggest that the decrease in the expression of these genes could be related to the inflammatory status of the oviduct, since some of these HSPs are related to inflammation and could elicit innate and adaptive proinflammatory immune responses [71,72]. In the same way, *HSPA4L* (mating (*q* < 0.05), SP-AI) and *HSP90AB1* (mating) expression, which was upregulated in the distal uterus, could be also related to inflammation, exerted by the mechanical stimulation produced by mating or the AI-catheter.

Our results also indicate the *STAT* genes (signal transducer and activator of transcription) may be important in the inflammation and also the transcription associated to cortisol action. These genes are present in the JAK/STAT pathway, which is the main signaling route for many cytokines [73]. In particular, *STAT5* has been shown to have important functions in reproduction and also in NR3C1-mediated transcriptional action [74,75], via active NR3C1-mediated transcription and T-cell differentiation [76]. Natural mating downregulated *STAT5A* in almost all tissues (ProxUt, DistUt, and Isth; *q* < 0.05) while *STAT5B* was only modified in the proximal uterus, and only by the sperm treatments (mating and semen-AI). Overall, these results may indicate that *STAT5A* downregulation, could be related to a reduction of the inflammatory response, which may in turn promote tolerance towards spermatozoa in the preovulatory tract, when spermatozoa have not participated in fertilization. Interestingly, a synergy between *STAT5A*, NF-κB, and *NR3C1* enhances *TLR2* expression [76], inducing an acute inflammatory response in the uterus that may remove sperm by activating polymorphonuclear neutrophil action in order to prepare an adequate implantation environment for the embryo in the female reproductive tract [77,78]. Other STAT proteins have been also related to immune and reproductive functions [74,79,80]. For example, *STAT3* is downregulated in the uterus and upregulated in the ampulla (*q* < 0.05), infundibulum, and in the UTJ (by seminal plasma only). The downregulation of *STAT3* in the uterus (and UTJ by SP-AI) may be also related to its role in the immune response, which is mediated by IL-6 and IL-10 [76], and recently shown to be associated with an increase in embryonic mortality in pigs after embryo transfer [81]. In addition, *STAT1* and *STAT2* key mediators of the innate immune response providing a first-line defense against pathogens [82], are downregulated by natural mating in the cervix and the uterus and might decrease the inflammation produced by semen deposition. *STAT6*, also downregulated after natural mating in the uterus, is consistent with the suppression of the innate immune response that we hypothesize for the rest of the STAT genes in this tissue.

Finally, natural mating produced in the sperm reservoir the same pattern of down (*HSD11B1, PTGS2, FKBP4,* and *STAT5A*) or upregulation (*NR3C1*, *HSD11B2,* and *FKBP5*) of genes directly involved in the glucocorticoid action. A decrease in GC availability and action could be beneficial in the sperm reservoir. Recent results in humans have found cortisol, testosterone, and other steroid-like molecules compete with progesterone binding to the sperm-membrane receptor, α/β hydrolase domain-containing protein 2 (ABHD2) [83]. This receptor, is responsible for removing inhibitors of the cation channel of sperm (CatSper), in an antagonist competition mechanism, consequently inhibiting hyperactivation [84]. Thus, GCs could be exerting an anti-capacitation effect by preventing premature CatSper activation occurring prior to ovulation, as well as preventing GCs from competing with progesterone, which is necessary for sperm hyperactivation, release from the reservoir oviductal epithelial cells [85], and chemotaxis [86]. Activation of CatSper channels by progesterone, or even prostaglandins, seems to differ among species [87]. We have previously shown that *ABHD2* is downregulated in the preovulatory UTJ and isthmus, perhaps preventing premature massive capacitation prior to ovulation [88].

## 4. Materials and Methods

### 4.1. Ethics Statement

Animal handling was performed conforming to current legislation of Sweden (SJVFS 2017:40) and European Community regulation (European Directive 2010/63/EU, 22/09/2010). The experimental research was previously approved by the “Regional Committee for Ethical Approval of Animal Experiments” (Linköpings Djurförsöksetiska nämnd) in Linköping, Sweden (permits no. 75–12 (10/02/2012) and no. ID1400, 02/02/2018).

### 4.2. Experimental Design of the Study

Sixteen domestic sows (*Sus scrofa domestica*) in the first day of spontaneous estrus were equally distributed in four groups: control (*n* = 4), the animals were infused cervically with 50 mL of Beltsville thawing solution (BTS) protein-free extender [89]; natural mating (*n* = 4), each sow was mated to a single boar; or cervical AI of either pools (5 boars) of the ejaculate sperm-rich first portion semen-AI, *n* = 4), or its sperm-free seminal plasma (SP-AI, *n* = 4) [90]. Tissue segments from the cervix (Cvx), distal uterus (DistUt), proximal uterus (ProxUt), utero-tubal junction (UTJ), isthmus (Isth), ampulla (Amp), and infundibulum (Inf) were surgically collected under general anesthesia, 24 h after the treatments, still preovulation, and subjected to gene expression analysis. 

### 4.3. Animal Management

Weaned sows (parity 1–3; *n* = 16) and mature boars (9–11 months old; *n* = 5) with good sperm quality (concentration, motility, and morphology) of Swedish Landrace breed were included as previously described [90]. Spontaneous 2nd estrus post-weaning was manually checked while sows held snout contact with a boar for standing reflex, upon which they were mated or alternatively intra-cervically inseminated using standard AI-catheters (Minitüb, Munich, Germany).

### 4.4. Collection and Handling of Semen and Tissue Samples

Ejaculates were collected weekly in individual fractions using the gloved-hand method and analyzed as previously described [90], to reach a pattern of regular ejaculates depicting >70% sperm motility and with >75% morphologically-normal spermatozoa. The ejaculate sperm-rich first portion [89] was used for the AI infusion in the semen-AI or SP-AI groups. Seminal plasma (SP-AI) was harvested through double centrifugation (1500 × *g*/ 10 min) and checked as sperm- and somatic cell-free. All sows were subjected to surgery (mid-laparotomy) under general anesthesia [89] to remove samples from the cervix, uterus, and oviduct. All samples were plunged into liquid nitrogen and later stored (−80 °C) until analyzed. Ovaries contained only un-ovulated follicles, in similar numbers among groups (22.30 ± 7.29, mean ± SD) and the condition of pre-ovulation was confirmed by the ratio of circulating estrogen:progesterone in blood plasma [89].

### 4.5. Microarray Hybridization and Scanning

Total RNA was extracted from tissue segments with Trizol using the protocols described elsewhere [27]. Complementary DNA (cDNA) of each sample was obtained from RNA (250 ng) of each sample by using the GeneChip® Whole Transcript Plus reagent kit (Affymetrix, Santa Clara, CA, USA) following the manufacturer protocol. The hybridization of cDNA mixture of each sample was made using labeled single-strand fragments of cDNA (3.5 μg; 41 μL) and hybridization master mix (109 μL). This cocktail was incubated at 99 °C for 5 min, decreasing the temperature to 45°C afterwards. The cocktail hybridization mix (130 μL) of each sample was then loaded on a microarray chip specific for porcine species (GeneChip® Porcine Gene 1.0 ST Array, ThermoFisher Scientific, Sweden) for incubation under rotation (60 revolutions per minute) at 45 °C for 16 h. After being incubated, the hybridized array was unloaded, washed, and stained (GeneChip® Fluidics Station 450, Affymetrix). The array chip was then scanned by using GeneChip® scanner GCS3000.

### 4.6. Microarray Data Analysis

All data obtained from each array was analyzed with the Transcriptome Analysis Console (TAC; version 4.0) from Affymetrix. Briefly, robust multi-array average (RMA) normalization, computing average intensity values by background adjustment, quantile normalization among arrays, and logarithmic transformation was performed in order to obtain the values of expression of each of the transcripts. Only genes related to the glucocorticoid receptor (NR3C1/GR) were assessed in detail. These 22 genes were reported as related to NR3C1 by using a combination of the internet-based tools. These interaction networks included protein and genetic interactions, pathways, and co-expression. Also, PANTHER (Protein Analysis Through Evolutionary Relationships) classification system for gene ontology (GO) [31] of biological process and molecular function was used for the analysis of the functions of the analyzed genes. Graphical illustration of overrepresented GO terms was produced with the Cytoscape v3.0.0 application CluePedia v2.0.3 [91]. Statistics of the normalized gene expression were determined using a linear model with an empirical Bayes method implemented in the specific package of Linear Models for Microarray Analysis (“Limma”). The statistical analyses were performed to detect differential expression of transcripts using a Benjamini−Hochberg false discovery rate (FDR) (*q* < 0.05) and a principal component analysis (PCA)-based p-value correction for type-I errors made with a statistical cut off *p* < 0.05 or FDR *q* < 0.05) [29], which was completed using ClustVis (BETA) web tool [92]. The principal component analysis clustered, after linear transformation, multivariate data ordered based on the variance. The prediction ellipses showed that the probability for a new observation from each group would be inside the ellipse (0.05 error). All the redundant or uncharacterized transcripts were excluded to obtain a final database of differentially-expressed genes. The list of genes found to be differentially expressed were then searched for functional pathways using the Kyoto Encyclopedia of Genes and Genomes (KEGG) database [30].

## 5. Conclusions

Overall, the results of this study indicate that natural mating seems to produce a differential response in the reproductive tract of the sow, compared to the use of AI, at least in the preovulatory phase. In porcine, it seems clear that in terms of effects on gene expression, some inherent effects of natural mating on GC regulation genes, could not be mimicked by AI. Moreover, the genes involved in the glucocorticoid receptor control (*FKBP4* and *FKBP5*), cortisol availability (*HSD11B1* and *HSD11B2*), and JAK-STAT signaling (*STAT5A*), exert a collective glucocorticoid-avoiding response that may prevent detrimental effects in the sperm reservoir and help sperm activation and detachment close to the time of ovulation.

## Supplementary Materials

Supplementary materials can be found at https://www.mdpi.com/1422-0067/21/12/4437/s1. Figure S1. Differentially expressed genes (DEGs) of cervix (Cvx), distal uterus (DistUt), proximal uterus (ProxUt), utero-tubal junction (UTJ), isthmus (Isth), ampulla (Amp) and infundibulum (Inf), ordered by treatments (natural mating, Semen-AI or SP-AI). Numbers represent the fold change for each gene on each tissue, compared to control. Upregulated genes (*p* < 0.05) are marked in green, while downregulated genes (*p* < 0.05) are shown in red colour. Colour grading is displayed ranging from 1 (upregulated), and from -1 (downregulated) in every treatment, separately. FDRs (*q* < 0.05) is noted in bold. Figure S2. Principal Component Analysis (PCA) of (A) proximal uterus (ProxUt), (B) uterotubal junction (UTJ) and (C) isthmus (Isth) was depicted. The prediction ellipses show the probability for a new observation from each group will be inside the ellipse (0.05 error). Principal component 1 (PC1) and principal component 2 (PC2) explain percentage of the total variance, respectively. Figure S3. Heat map of (A) proximal uterus (ProxUt), (B) utero-tubal junction (UTJ) and (C) isthmus (Isth) was depicted. Data was clustered using correlation distance and average linkage. Table S1. Kyoto Encyclopedia of Gens and Genomes (KEGG) Pathways of genes differentially expressed by natural mating. Upregulation is represented in bold and downregulation in non-bold (*p* < 0.05) in each tissue: cervix (Cvx), distal uterus (DistUt), proximal uterus (ProxUt), utero-tubal junction (UTJ), isthmus (Isth), ampulla (Amp) and infundibulum (Inf). Also, false discovery rates (FDRs) are shown in red (*q* < 0.05). Table S2. KEGG Pathways of genes differentially expressed by cervical insemination of the first portion of the sperm-rich ejaculate fraction (Semen-AI). Upregulation is shown in bold and downregulation in non-bold (*p* < 0.05) in each tissue: cervix (Cvx), distal uterus (DistUt), proximal uterus (ProxUt), utero-tubal junction (UTJ), isthmus (Isth), ampulla (Amp) and infundibulum (Inf). Table S3. KEGG Pathways of genes differentially expressed by cervical insemination of the sperm-free seminal plasma of the first portion of the sperm-rich fraction (SP-AI). Upregulation is shown in bold and downregulation in non-bold (*p* < 0.05) in each tissue: cervix (Cvx), distal uterus (DistUt), proximal uterus (ProxUt), utero-tubal junction (UTJ), isthmus (Isth), ampulla (Amp) and infundibulum (Inf).
