# Supplementary material for: Natural Mating Differentially Triggers Expression of Glucocorticoid Receptor (NR3C1)-Related Genes in the Preovulatory Porcine Female Reproductive Tract
Source: Int J Mol Sci. 2020 Jun 22;21(12):4437. doi: 10.3390/ijms21124437 (PMC7352215; doi:10.3390/ijms21124437)
Supplement: Supplementary file 1 [file ijms-21-04437-s001.pdf]

	MATING							SEMEN-AI							SP-AI						
	Cvx	DistUt	ProxUt	UTJ	Isth	Amp	Inf	Cvx	DistUt	ProxUt	UTJ	Isth	Amp	Inf	Cvx	DistUt	ProxUt	UTJ	Isth	Amp	Inf
NR3C1					1,3		1,35							1,28					1,35		
HSD11B1	-2,55	**-3,92**	**-3,87**	-2,69	-1,96																
HSD11B2					1,35	1,38	**1,71**			-1,56											
FKBP5		1,5	2,07	1,98	2,36																
FKBP4	-2,22	-1,37	-1,4	**-2**	**-2,73**	**-2,2**	**-1,85**			-1,26		-1,24		-1,34							
PTGS1			1,2			1,3			-1,33							-1,3					
PTGS2	-3,37		-2,06	-2,23	-2,01														1,7		
PLA2G4B						1,34							1,3	1,19						1,29	
IGFBP1																					
HSPA8						-1,26	-1,37				-1,37	-1,52	-1,36	-1,28							
STAT1	-1,66	-1,64	-1,52										-1,26							-1,13	
STAT2	-1,69	-1,67	-1,65																		
STAT3		-1,56	-1,92			**1,44**	1,26											-1,47			
STAT5A	-1,43	**-2,5**	**-2,38**	-1,5	**-1,5**		-1,28														-1,33
STAT5B			-1,3							-1,31											
STAT6		-1,23				1,31	**1,39**						1,26								
TP53	-1,17				-1,17														-1,29		-1,37
MED1						-1,13							-1,16								
MED14						-1,1	-1,21														
HSPA4	-1,45												-1,34	-1,21							
HSPA4L		**1,58**				-1,67	-2,02	-2,25			-1,48					1,8					
HSP90AB1		1,18				-1,26	-1,17														

Supplementary figure 1. Differentially expressed genes (DEGs) of cervix (Cvx), distal uterus (DistUt), proximal uterus (ProxUt), utero-tubal junction (UTJ), isthmus (Isth), ampulla (Amp) and infundibulum (Inf), ordered by treatments (natural mating, Semen-AI or SP-AI). Numbers represent the fold change for each gene on each tissue, compared to control. Upregulated genes ($p<0.05$) are marked in green, while downregulated genes ($p<0.05$) are shown in red colour. Colour grading is displayed ranging from 1 (upregulated), and from -1 (downregulated) in every treatment, separately. FDRs ($q<0.05$) is noted in bold.

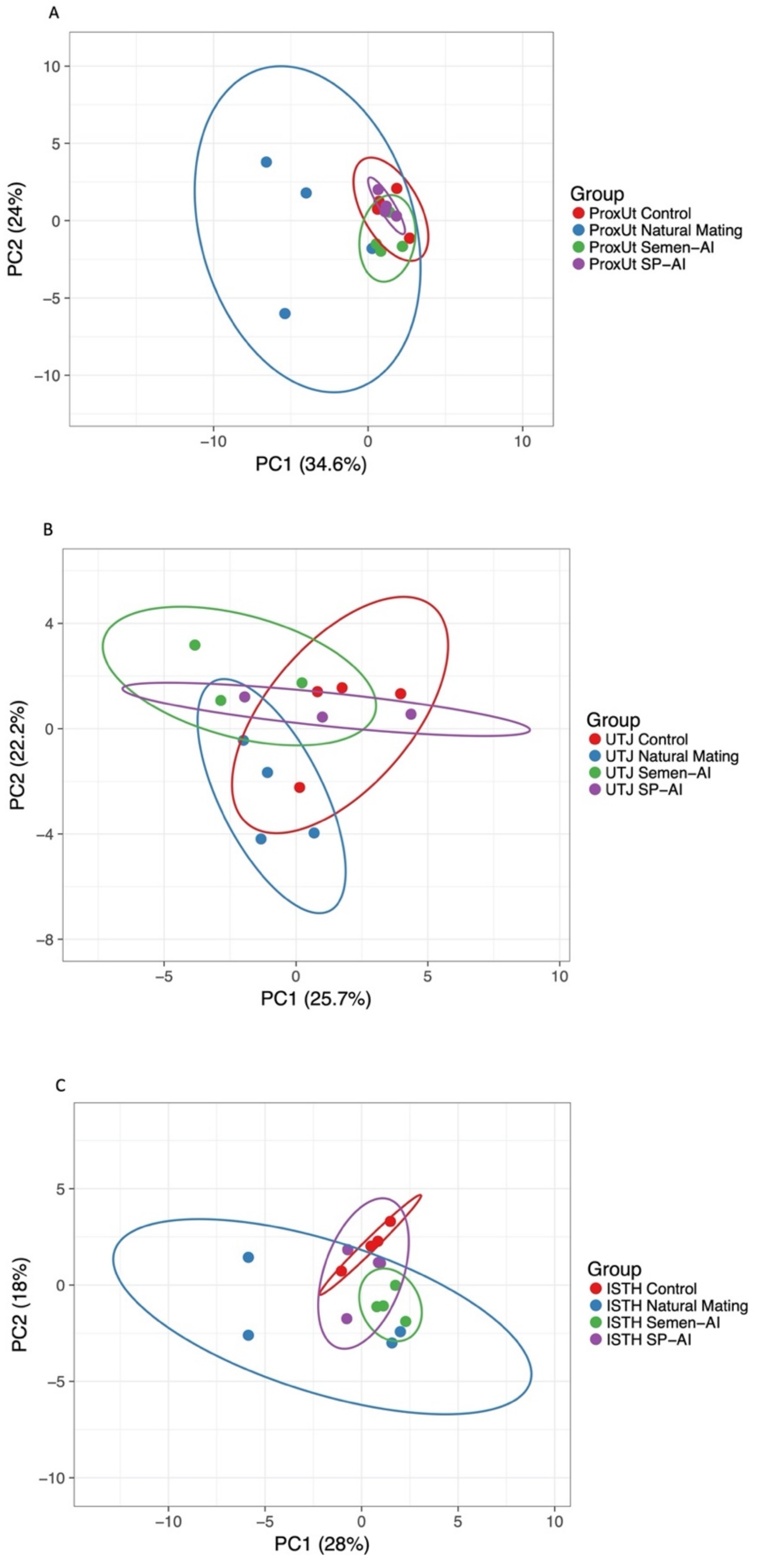

Supplementary Figure 2. Principal Component Analysis (PCA) of A) proximal uterus (ProxUt), B) utero-tubal junction (UTJ) and C) isthmus (Isth) was depicted. The prediction ellipses show the probability for a new observation from each group will be inside the ellipse (0.05 error). Principal component 1 (PC1) and principal component 2 (PC2) explain percentage of the total variance, respectively.

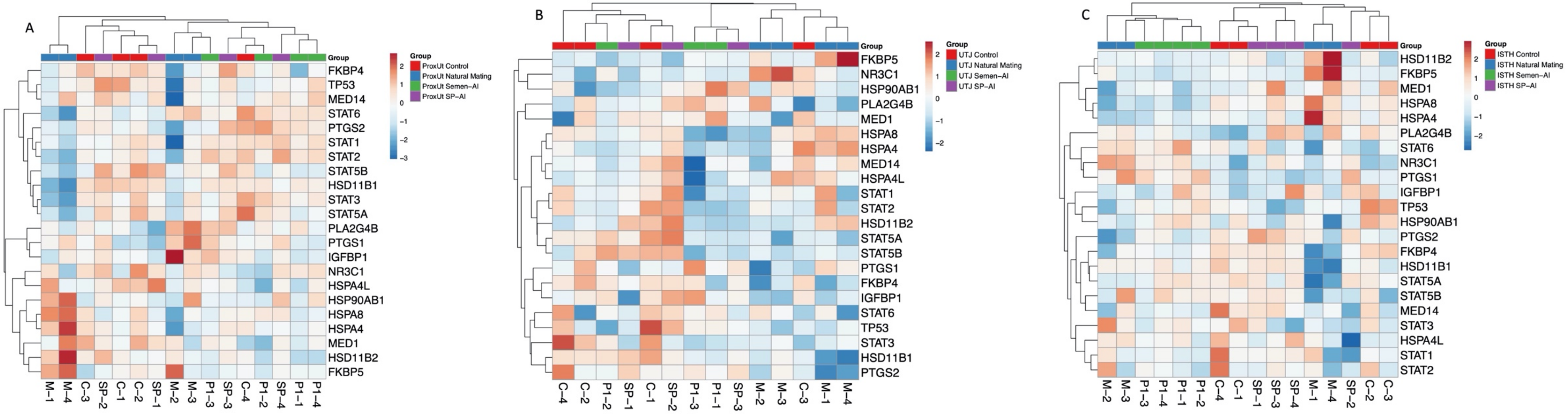

Supplementary Figure 3. Heat map of A) proximal uterus (ProxUt), B) utero-tubal junction (UTJ) and C) isthmus (Isth) was depicted. Data was clustered using correlation distance and average linkage.

Supplementary Table 1. Kyoto Encyclopedia of Gens and Genomes (KEGG) Pathways of genes differentially expressed by natural mating. Upregulation is represented in bold and downregulation in non-bold ($p<0.05$) in each tissue: cervix (Cvx), distal uterus (DistUt), proximal uterus (ProxUt), utero-tubal junction (UTJ), isthmus (Isth), ampulla (Amp) and infundibulum (Inf). Also, false discovery rates (FDRs) are shown in red ($q<0.05$).

**Natural Mating**		
**Gene List**	**Tissue**	**Pathway Name & ID**
NR3C1	**Isth**, **Inf**	Neuroactive ligand-receptor interaction (ssc04080)
HSD11B1	Cvx, DistUt, ProxUt, UTJ, Isth	Steroid hormone biosynthesis (ssc00140)<br>Metabolism of xenobiotics by cytochrome P450 (ssc00980)<br>Metabolic pathways (ssc01100)
HSD11B2	**Isth, Amp, Inf**	Steroid hormone biosynthesis (ssc00140)<br>Metabolic pathways (ssc01100)<br>Aldosterone-regulated sodium reabsorption (ssc04960)
FKBP5	**DistUt, ProxUt, UTJ, Isth**	Estrogen signaling pathway (ssc04915)
PTGS1	**ProxUt, Amp**	Arachidonic acid metabolism (ssc00590)<br>Metabolic pathways (ssc01100)<br>Platelet activation (ssc04611)<br>Serotonergic synapse (ssc04726)<br>Regulation of lipolysis in adipocytes (ssc04923)
PTGS2	Cvx, DistUt, UTJ, **Isth**	Arachidonic acid metabolism (ssc00590)<br>Metabolic pathways (ssc01100)<br>NF-kappa B signaling pathway (ssc04064)<br>VEGF signaling pathway (ssc04370)<br>C-type lectin receptor signaling pathway (ssc04625)<br>IL-17 signaling pathway (04657)<br>TNF signaling pathway (ssc04668)<br>Retrograde endocannabinoid signaling (ssc04723)<br>Serotonergic synapse (ssc04726)<br>Ovarian steroidogenesis (ssc04913)<br>Oxytocin signaling pathway (ssc04921)<br>Regulation of lipolysis in adipocytes (ssc04923)
PLA2G4B	**Amp**	Glycerophospholipid metabolism (ssc00564)<br>Ether lipid metabolism (ssc00565)<br>Arachidonic acid metabolism (ssc00590)<br>Linoleic acid metabolism (ssc00591)<br>alpha-Linolenic acid metabolism (ssc00592)<br>Metabolic pathways (ssc01100)<br>MAPK signaling pathway (ssc04010)<br>Ras signaling pathway (ssc04014)<br>Phospholipase D signaling pathway (ssc04072)<br>Necroptosis (ssc04217)<br>Vascular smooth muscle contraction (ssc04270)<br>VEGF signaling pathway (ssc04370)<br>Platelet activation (ssc04611)<br>Fc epsilon RI signaling pathway (ssc04664)<br>Fc gamma R-mediated phagocytosis (ssc04666)<br>Glutamatergic synapse (ssc04724)<br>Serotonergic synapse (ssc04726)<br>Long-term depression (ssc04730)<br>Inflammatory mediator regulation of TRP channels (ssc04750)<br>GnRH signaling pathway (ssc04912)<br>Ovarian steroidogenesis (ssc04913)<br>Oxytocin signaling pathway (ssc04921)<br>Choline metabolism in cancer (ssc05231)

HSPA8	Amp, Inf	Spliceosome (ssc03040)<br>MAPK signaling pathway (ssc04010)<br>Protein processing in endoplasmic reticulum (ssc04141)<br>Endocytosis (ssc04144)<br>Longevity regulating pathway - multiple species (ssc04213)<br>Antigen processing and presentation (ssc04612)<br>Estrogen signaling pathway (ssc04915)
FKBP4	Cvx, DistUt, ProxUt, UTJ, Isth, Amp, Inf	Estrogen signaling pathway (ssc04915)
STAT1	Cvx, DistUt, ProxUt	Chemokine signaling pathway (ssc04062)<br>Necroptosis (ssc04217)<br>Toll-like receptor signaling pathway (ssc04620)<br>NOD-like receptor signaling pathway (ssc04621)<br>C-type lectin receptor signaling pathway (ssc04625)<br>Jak-STAT signaling pathway (ssc04630)<br>Th1 and Th2 cell differentiation (ssc04658)<br>Th17 cell differentiation (ssc04659)<br>Prolactin signaling pathway (ssc04917)<br>Thyroid hormone signaling pathway (ssc04919)<br>AGE-RAGE signaling pathway in diabetic complications (ssc04933)<br>Growth hormone synthesis, secretion and action (ssc04935)
STAT2	Cvx, DistUt, ProxUt	Chemokine signaling pathway (ssc04062)<br>Necroptosis (ssc04217)<br>NOD-like receptor signaling pathway (ssc04621)<br>C-type lectin receptor signaling pathway (ssc04625)<br>Jak-STAT signaling pathway (ssc04630)
STAT3	DistUt, ProxUt, **Amp**, **Inf**	EGFR tyrosine kinase inhibitor resistance (ssc01521)<br>Chemokine signaling pathway (ssc04062)<br>HIF-1 signaling pathway (ssc04066)<br>FoxO signaling pathway (ssc04068)<br>Necroptosis (ssc04217)<br>Signaling pathways regulating pluripotency of stem cells (ssc04550)<br>Jak-STAT signaling pathway (ssc04630)<br>Th17 cell differentiation (ssc04659)<br>Prolactin signaling pathway (ssc04917)<br>Adipocytokine signaling pathway (ssc04920)<br>Insulin resistance (ssc04931)<br>AGE-RAGE signaling pathway in diabetic complications (ssc04933)<br>Growth hormone synthesis, secretion and action (ssc04935)
STAT5A	Cvx, DistUt, ProxUt, UTJ, Isth, Inf	ErbB signaling pathway (ssc04012)<br>Necroptosis (ssc04217)<br>Jak-STAT signaling pathway (ssc04630)<br>Th1 and Th2 cell differentiation (ssc04658)<br>Th17 cell differentiation (ssc04659)<br>Prolactin signaling pathway (ssc04917)<br>AGE-RAGE signaling pathway in diabetic complications (ssc04933)<br>Growth hormone synthesis, secretion and action (ssc04935)
STAT5B	**ProxUt**	ErbB signaling pathway (ssc04012)<br>Chemokine signaling pathway (ssc04062)<br>Necroptosis (ssc04217)<br>Jak-STAT signaling pathway (ssc04630)<br>Th1 and Th2 cell differentiation (ssc04658)<br>Th17 cell differentiation (ssc04659)<br>Prolactin signaling pathway (ssc04917)<br>AGE-RAGE signaling pathway in diabetic complications (ssc04933)<br>Growth hormone synthesis, secretion and action (ssc04935)
STAT6	DistUt, **Amp**, **Inf**	Necroptosis (ssc04217)<br>Jak-STAT signaling pathway (ssc04630)<br>Th1 and Th2 cell differentiation (ssc04658)<br>Th17 cell differentiation (ssc04659)

TP53	Cvx, Isth	Endocrine resistance (ssc01522)<br>Platinum drug resistance (ssc01524)<br>MAPK signaling pathway (ssc04010)<br>Sphingolipid signaling pathway (ssc04071)<br>Cell cycle (ssc04110)<br>p53 signaling pathway (ssc04115)<br>Mitophagy – animal (ssc04137)<br>PI3K-Akt signaling pathway (ssc04151)<br>Apoptosis (ssc04210)<br>Longevity regulating pathway (ssc04211)<br>Ferroptosis (ssc04216)<br>Cellular senescence (ssc04218)<br>Wnt signaling pathway (ssc04310)<br>Neurotrophin signaling pathway (ssc04722)<br>Thyroid hormone signaling pathway (ssc04919)
MED1	Amp	Endocrine resistance (ssc01522)<br>Thyroid hormone signaling pathway (ssc04919)
MED14	Amp, Inf	Thyroid hormone signaling pathway (ssc04919)
HSPA4	Cvx	Tight junction (ssc04530)<br>Antigen processing and presentation (ssc04612)
HSPA4L	**DistUt**, Amp, Inf	Protein processing in endoplasmic reticulum (ssc04141)
HSP90AB1	**DistUt**, Amp, Inf	Protein processing in endoplasmic reticulum (ssc04141)<br>PI3K-Akt signaling pathway (ssc04151)<br>Necroptosis (ssc04217)<br>Antigen processing and presentation (ssc04612)<br>NOD-like receptor signaling pathway (ssc04621)<br>IL-17 signaling pathway (ssc04657)<br>Th17 cell differentiation (ssc04659)<br>Progesterone-mediated oocyte maturation (ssc04914)<br>Estrogen signaling pathway (ssc04915)

Supplementary Table 2. KEGG Pathways of genes differentially expressed by cervical insemination of the first portion of the sperm-rich ejaculate fraction (Semen-AI). Upregulation is shown in bold and downregulation in non-bold ($p<0.05$) in each tissue: cervix (Cvx), distal uterus (DistUt), proximal uterus (ProxUt), utero-tubal junction (UTJ), isthmus (Isth), ampulla (Amp) and infundibulum (Inf).

**Semen-AI**		
**Gene List**	**Tissue**	**Pathway Name & ID**
NR3C1	**Inf**	Neuroactive ligand-receptor interaction (ssc04080)
HSD11B2	ProxUt	Steroid hormone biosynthesis (ssc00140)<br>Metabolic pathways (ssc01100)<br>Aldosterone-regulated sodium reabsorption (ssc04960)
PTGS1	DistUt	Arachidonic acid metabolism (ssc00590)<br>Metabolic pathways (ssc01100)<br>Platelet activation (ssc04611)<br>Serotonergic synapse (ssc04726)<br>Regulation of lipolysis in adipocytes (ssc04923)
PLA2G4B	**Amp, Inf**	Glycerophospholipid metabolism (ssc00564)<br>Ether lipid metabolism (ssc00565)<br>Arachidonic acid metabolism (ssc00590)<br>Linoleic acid metabolism (ssc00591)<br>alpha-Linolenic acid metabolism (ssc00592)<br>Metabolic pathways (ssc01100)<br>MAPK signaling pathway (ssc04010)<br>Ras signaling pathway (ssc04014)<br>Phospholipase D signaling pathway (ssc04072)<br>Necroptosis (ssc04217)<br>Vascular smooth muscle contraction (ssc04270)<br>VEGF signaling pathway (ssc04370)<br>Platelet activation (ssc04611)<br>Fc epsilon RI signaling pathway (ssc04664)<br>Fc gamma R-mediated phagocytosis (ssc04666)<br>Glutamatergic synapse (ssc04724)<br>Serotonergic synapse (ssc04726)<br>Long-term depression (ssc04730)<br>Inflammatory mediator regulation of TRP channels (ssc04750)<br>GnRH signaling pathway (ssc04912)<br>Ovarian steroidogenesis (ssc04913)<br>Oxytocin signaling pathway (ssc04921)<br>Choline metabolism in cancer (ssc05231)
HSPA8	UTJ, Isth, Amp, Inf	Spliceosome (ssc03040)<br>MAPK signaling pathway (ssc04010)<br>Protein processing in endoplasmic reticulum (ssc04141)<br>Endocytosis (ssc04144)<br>Longevity regulating pathway - multiple species (ssc04213)<br>Antigen processing and presentation (ssc04612)<br>Estrogen signaling pathway (ssc04915)
FKBP4	ProxUt, Isth, Inf	Estrogen signaling pathway (ssc04915)
STAT1	Amp	Chemokine signaling pathway (ssc04062)<br>Necroptosis (ssc04217)<br>Toll-like receptor signaling pathway (ssc04620)<br>NOD-like receptor signaling pathway (ssc04621)<br>C-type lectin receptor signaling pathway (ssc04625)<br>Jak-STAT signaling pathway (ssc04630)<br>Th1 and Th2 cell differentiation (ssc04658)<br>Th17 cell differentiation (ssc04659)<br>Prolactin signaling pathway (ssc04917)

		Thyroid hormone signaling pathway (ssc04919)AGE-RAGE signaling pathway in diabetic complications (ssc04933)<br>Growth hormone synthesis, secretion and action (ssc04935)
STAT5B	ProxUt	ErbB signaling pathway (ssc04012)<br>Chemokine signaling pathway (ssc04062)<br>Necroptosis (ssc04217)<br>Jak-STAT signaling pathway (ssc04630)<br>Th1 and Th2 cell differentiation (ssc04658)<br>Th17 cell differentiation (ssc04659)<br>Prolactin signaling pathway (ssc04917)<br>AGE-RAGE signaling pathway in diabetic complications (ssc04933)<br>Growth hormone synthesis, secretion and action (ssc04935)
STAT6	**Amp**	Necroptosis (ssc04217)<br>Jak-STAT signaling pathway (ssc04630)<br>Th1 and Th2 cell differentiation (ssc04658)<br>Th17 cell differentiation (ssc04659)
MED1	Amp	Endocrine resistance (ssc01522)<br>Thyroid hormone signaling pathway (ssc04919)
HSPA4	Amp, Inf	Tight junction (ssc04530)<br>Antigen processing and presentation (ssc04612)
HSPA4L	Cvx, UTJ	Protein processing in endoplasmic reticulum (ssc04141)

Supplementary Table 3. KEGG Pathways of genes differentially expressed by cervical insemination of the sperm-free seminal plasma of the first portion of the sperm-rich fraction (SP-AI). Upregulation is shown in bold and downregulation in non-bold ($p<0.05$) in each tissue: cervix (Cvx), distal uterus (DistUt), proximal uterus (ProxUt), utero-tubal junction (UTJ), isthmus (Isth), ampulla (Amp) and infundibulum (Inf).

SP-AI

Gene List	Tissue	Pathway Name & ID
NR3C1	**Isth**	Neuroactive ligand-receptor interaction (ssc04080)
PTGS1	DistUt	Arachidonic acid metabolism (ssc00590)<br>Metabolic pathways (ssc01100)<br>Platelet activation (ssc04611)<br>Serotonergic synapse (ssc04726)<br>Regulation of lipolysis in adipocytes (ssc04923)
PTGS2	**Isth**	Arachidonic acid metabolism (ssc00590)<br>Metabolic pathways (ssc01100)<br>NF-kappa B signaling pathway (ssc04064)<br>VEGF signaling pathway (ssc04370)<br>C-type lectin receptor signaling pathway (ssc04625)<br>IL-17 signaling pathway (04657)<br>TNF signaling pathway (ssc04668)<br>Retrograde endocannabinoid signaling (ssc04723)<br>Serotonergic synapse (ssc04726)<br>Ovarian steroidogenesis (ssc04913)<br>Oxytocin signaling pathway (ssc04921)<br>Regulation of lipolysis in adipocytes (ssc04923)
PLA2G4B	**Amp**	Glycerophospholipid metabolism (ssc00564)<br>Ether lipid metabolism (ssc00565)<br>Arachidonic acid metabolism (ssc00590)<br>Linoleic acid metabolism (ssc00591)<br>alpha-Linolenic acid metabolism (ssc00592)<br>Metabolic pathways (ssc01100)<br>MAPK signaling pathway (ssc04010)<br>Ras signaling pathway (ssc04014)<br>Phospholipase D signaling pathway (ssc04072)<br>Necroptosis (ssc04217)<br>Vascular smooth muscle contraction (ssc04270)<br>VEGF signaling pathway (ssc04370)<br>Platelet activation (ssc04611)<br>Fc epsilon RI signaling pathway (ssc04664)<br>Fc gamma R-mediated phagocytosis (ssc04666)<br>Glutamatergic synapse (ssc04724)<br>Serotonergic synapse (ssc04726)<br>Long-term depression (ssc04730)<br>Inflammatory mediator regulation of TRP channels (ssc04750)<br>GnRH signaling pathway (ssc04912)<br>Ovarian steroidogenesis (ssc04913)<br>Oxytocin signaling pathway (ssc04921)<br>Choline metabolism in cancer (ssc05231)
STAT1	Amp	Chemokine signaling pathway (ssc04062)<br>Necroptosis (ssc04217)<br>Toll-like receptor signaling pathway (ssc04620)<br>NOD-like receptor signaling pathway (ssc04621)<br>C-type lectin receptor signaling pathway (ssc04625)<br>Jak-STAT signaling pathway (ssc04630)<br>Th1 and Th2 cell differentiation (ssc04658)<br>Th17 cell differentiation (ssc04659)<br>Prolactin signaling pathway (ssc04917)<br>Thyroid hormone signaling pathway (ssc04919)

STAT3	UTJ	EGFR tyrosine kinase inhibitor resistance (ssc01521)<br>Chemokine signaling pathway (ssc04062)<br>HIF-1 signaling pathway (ssc04066)<br>FoxO signaling pathway (ssc04068)<br>Necroptosis (ssc04217)<br>Signaling pathways regulating pluripotency of stem cells (ssc04550)<br>Jak-STAT signaling pathway (ssc04630)<br>Th17 cell differentiation (ssc04659)<br>Prolactin signaling pathway (ssc04917)<br>Adipocytokine signaling pathway (ssc04920)<br>Insulin resistance (ssc04931)<br>AGE-RAGE signaling pathway in diabetic complications (ssc04933)<br>Growth hormone synthesis, secretion and action (ssc04935)
STAT5A	Inf	ErbB signaling pathway (ssc04012)<br>Necroptosis (ssc04217)<br>Jak-STAT signaling pathway (ssc04630)<br>Th1 and Th2 cell differentiation (ssc04658)<br>Th17 cell differentiation (ssc04659)<br>Prolactin signaling pathway (ssc04917)<br>AGE-RAGE signaling pathway in diabetic complications (ssc04933)<br>Growth hormone synthesis, secretion and action (ssc04935)
TP53	Isth, Inf	Endocrine resistance (ssc01522)<br>Platinum drug resistance (ssc01524)<br>MAPK signaling pathway (ssc04010)<br>Sphingolipid signaling pathway (ssc04071)<br>Cell cycle (ssc04110)<br>p53 signaling pathway (ssc04115)<br>Mitophagy – animal (ssc04137)<br>PI3K-Akt signaling pathway (ssc04151)<br>Apoptosis (ssc04210)<br>Longevity regulating pathway (ssc04211)<br>Ferroptosis (ssc04216)<br>Cellular senescence (ssc04218)<br>Wnt signaling pathway (ssc04310)<br>Neurotrophin signaling pathway (ssc04722)<br>Thyroid hormone signaling pathway (ssc04919)
HSPA4L	**DistUt**	Protein processing in endoplasmic reticulum (ssc04141)
